# Development of Nanofluids for the Inhibition of Formation Damage Caused by Fines Migration: Effect of the Interaction of Quaternary Amine (CTAB) and MgO Nanoparticles

**DOI:** 10.3390/nano10050928

**Published:** 2020-05-11

**Authors:** Rebeka Díez, Oscar E. Medina, Lady J. Giraldo, Farid B. Cortés, Camilo A. Franco

**Affiliations:** Research Group on Surface Phenomena - Michael Polanyi, Faculty of Mines, National University of Colombia, 050034 Medellín, Colombia; rdiezb@unal.edu.co (R.D.); oemedinae@unal.edu.co (O.E.M.); ljgiraldop@unal.edu.co (L.J.G.)

**Keywords:** CTAB, fines migration, formation damage, MgO nanoparticles, nanocomposite

## Abstract

Fines migration is a common problem in the oil and gas industry that causes a decrease in productivity. In this sense, the main objective of this study is to develop nanocomposites based on the interaction of quaternary amine (hexadecyltrimethylammonium bromide—CTAB) and MgO to enhance the capacity of retention of fine particles in the porous medium. MgO nanoparticles were synthesized by the sol–gel method using Mg(NO_3_)_2_·6H_2_O as a precursor. Nanoparticles were characterized by dynamic light scattering (DLS), the point of zero charge (pH_pzc_), thermogravimetric analysis, and Fourier transform infrared spectroscopy (FT-IR). Different nanoparticle sizes of 11.4, 42.8, and 86.2 nm were obtained, which were used for preparing two system nanofluids. These systems were evaluated in the inhibition of fines migration: in the system I MgO nanoparticles were dispersed in a CTAB-containing aqueous solution, and system II consists of a nanocomposite of CTAB adsorbed onto MgO nanoparticles. The fines retention tests were performed using Ottawa sand 20/40 packed beds and fine particles suspensions at concentrations of 0.2% in a mass fraction in deionized water. Individual and combined effects of nanoparticles and CTAB were evaluated in different treatment dosages. The analysis of the interactions between the CTAB and the MgO nanoparticles was carried out through batch-mode adsorption and desorption tests. The best treatment in the system I was selected according to the fines retention capacity and optimized through a simplex-centroid mixture design for mass fractions from 0.0% to 2.0% of both CTAB and MgO nanoparticles. This statistical analysis shows that the optimal concentration of these components is reached for a mass fraction of 0.73% of MgO nanoparticles and 0.74% in mass fraction of CTAB, where the retention capacity of the porous medium increases from 0.02 to 0.39 mg·L^−1^. Based on the experimental results, the nanofluids combining both components showed higher retention of fines than the systems treated only with CTAB or with MgO nanoparticles, with efficiencies up to 400% higher in the system I and higher up to 600% in the system II. To evaluate the best performance treatment under reservoir conditions, there were developed core flooding tests at fixed overburden pressure of 34.5 MPa, pore pressure at 6.9 MPa and system temperature at 93 °C. Obtaining critical rate increases in 142.8%, and 144.4% for water and oil flow in the presence of the nanofluid. In this sense, this work offers a new alternative for the injection of nanocomposites as a treatment for the problem of fines migration to optimize the productivity of oil and gas wells.

## 1. Introduction

In the oil and gas industry, there are significant problems related to formation damage during oil production, which cause substantial financial losses due to the reduction of well productivity and injectivity [1,2,3,4]. In particular, the transport of fine particles is one of the primary mechanisms contributing to the skin parameter in oil and gas wells, mainly in reservoirs with low permeability and small pore sizes [1,5]. These solids may be mineral fractions present in the porous medium such as clays, amorphous silica, quartz, feldspars, micas, and carbonates [1], or external solids that get into the formation with the injection of drilling fluids or other fluids used during the field operations [6,7].

The generation of fines migration inside the reservoir is related to different factors such as salinity, pH changes, wettability, the concentration of fine particles, flow rate, the ionic strength of the medium, and fractional flow of water and oil [6,8,9,10]. Therefore, the interaction forces between the porous media and the fine particles are determined by the surface alteration caused by colloidal forces (attractive Van der Waals forces, Born repulsive forces, and electrostatic interactions between the double electric layer inherent to each particle), and hydrodynamic forces [8,10].

Hence, different techniques and treatments have been used to overcome the above issues, such as matrix acidizing treatments [11,12] or the application of chemical stabilizers [13,14]. Nevertheless, these treatments could have different disadvantages, including many mixed reactions with hazardous substances and the possibility of generating another source of formation damage [11,13,15,16].

Another mechanism used to remediate the formation damage is changing the surface charges of the porous media through a zeta potential altering system [17,18]; for this, some amine-based cationic surfactants [19] and some additives [20,21], such as potassium chloride, ammonium chloride, and tetramethylammonium chloride, have been studied because of their capacity to bind to the clay surface by exchanging cations with its layer resulting in its integration and neutralizing the negative charges on the clay surface [19]. Notably, the cetyl trimethyl ammonium bromide (CTAB) has been used due to the quaternary ammonium end, which provides to the porous media the positive charges, while the hydrocarbon chains supply a steric exclusion effect [22].

Another current option that has shown promising results under laboratory is the use of nanoparticles and nanofluids of different chemical nature such as silicon oxide (SiO_2_) [23,24,25,26], aluminum oxide (Al_2_O_3_) [24,25,27], magnesium oxide (MgO) [26,27,28], iron oxide (Fe_2_O_3_), nickel oxide (NiO) [29], and hydrophobic silicon oxide (SiO_2_H) [30], among others. Due to their nanometric size (1–100 nm), nanoparticles are suitable for injection into the medium without risk of plugging near the well face. Additionally, nanoparticles allow the stabilization of the fine particles through surface interaction forces and inhibit the damage caused by the movement of the solids [31]. The change in the surface forces attributed to nanoparticles demonstrated the ability to trap the fines when they migrate from the rock surface [32], even leading to increases in the productivity, extending the production life of the well and minimizing the frequency of intervention as proved in field applications [32,33,34]. For example, in Colombia, it was developed a field test with a silica-based nanofluid. The results showed an increase in production after the nanofluid application. The increase of 48 bbl of oil compared to the baseline and the increase of 134 bbl relative to the previous treatment stage, show the optimum behavior of the nanoparticles. Additionally, the production of gas increased by 1000 Kscfd compared to the baseline. This demonstrated the effectiveness of the treatment in the retention of fine particles in the treated formation [35].

However, among the nanoparticles of different chemical natures that have been evaluated, the MgO nanoparticles have shown the best behavior on the retention of fine particles due to the total energy of the interaction is mainly attractive when dealing with sandstone reservoirs [27,36]. However, in the specialized literature, there are no reports of nanofluids based on the interaction of quaternary amines and MgO applied to the inhibition of the formation damage due to fines migration.

Therefore, the main objective of this study is to develop nanofluids based on the interaction of CTAB and MgO nanoparticles that allow a synergistic effect on the retention of migrating fine particles. Here, two systems were evaluated. The system I consisted of MgO nanoparticles dispersed in a CTAB-containing aqueous solution. In this system, nanoparticles with surfactant adsorbed coexist with free surfactant in solution. The system I was optimized for fines retention through a design of experiments with mixtures by varying the dosage of both CTAB and nanoparticles. From this optimization, the System II is proposed and consists of the evaluation of a nanocomposite composed of MgO nanoparticles with anchored CTAB on the surface. Here, the amount of free surfactant is null. For the nanofluid formulation, the nanocomposite is dispersed in water, i.e., there is no initially free surfactant remaining in the solution. This document is divided into three main sections including (i) the evaluation of MgO nanoparticles-based nanofluids composed of nanoparticles dispersed in a CTAB surfactant solution at different concentrations, (ii) the nanocomposite evaluation in the absence of free surfactant in the solution to assess the synergistic effect between the MgO support and the quaternary amine, and (iii) the core flooding test to evaluate fines migration inhibition at reservoir conditions in the absence and presence of an optimized nanofluid containing MgO nanoparticles with CTAB. The best nanocomposite was selected according to the amount of CTAB adsorbed over the MgO nanoparticles surface and optimized through a simplex-centroid mixture design (SCMD) of experiments.

## 2. Materials and Methods

### 2.1. Materials

Magnesium nitrate hexahydrate (MgNO_3_·6H_2_O, 98.0%, Panreac, Barcelona, Spain), sodium hydroxide (NaOH, 98.0%, Panreac, Barcelona, Spain), and methanol (CH_3_OH, 99.9%, Panreac, Barcelona, Spain) were used to synthesize the MgO nanoparticles. Hexadecyltrimethylammonium bromide (CTAB, 98.0%, Panreac, Barcelona, Spain) and deionized water were employed to synthesize the proposed treatments. Silica sand (Ottawa sand, US sieves 20−40 mesh, Minercol S.A.S, Bogotá D.C., Colombia) was cleaned with deionized water and used for porous medium preparation. The fines suspension was prepared using 0.2% in mass fraction of kaolinite (240 Mesh, Minercol S.A.S, Bogotá D.C., Colombia) particles in deionized water, considering the concentration of some Colombian fields [35]. Besides, hydrochloric acid (HCl, 37%, Sigma Aldrich, St. Louis, MO, USA) and sodium hydroxide (NaOH, 98.0%, Panreac, Barcelona, Spain) dilutions were employed to generate pH changes in the zeta potential measurements. Finally, potassium bromide (KBr, Pike technologies, Fitchburg, WI, USA) pellets were used for Fourier transform infrared spectroscopy (FT-IR) test.

A light crude oil (LO) from a Colombian field was employed for the displacement tests. The selected LO has an American Petroleum Institute (API) gravity of 33.1°, a viscosity of 7.55 cP at 25 °C, and a saturate, aromatic, resin and asphaltene content of 58.93%, 26.47%, 14.27%, and 0.30% by in mass fraction.

### 2.2. Methods

#### 2.2.1. Synthesis of MgO Nanoparticles

The MgO nanoparticles were synthesized through the sol–gel method according to the procedure proposed by Wahab et al. [37]. The sol–gel method consists of the gelation of a solution of colloids where the particles dispersed in the solution are commonly known as sols that at defined conditions of temperature and pH form the gel [38]. For the sols formation, magnesium nitrate hexahydrate was used as a magnesium precursor in a deionized water solution. The solution is continuously stirred at 500 rpm while a 0.5 M NaOH solution is added dropwise until a pH value of 12.5 is reached. The magnesium nitrate hexahydrate/sodium hydroxide molar ratio was varied in 0.4, 0.8, and 1.2 to obtain different particle sizes.

Further, a white precipitate is obtained by filtration from the solution and washed with methanol to remove ionic impurities. Finally, the precipitate was dried at 70 °C and later calcined at 400 °C for two hours to obtain the MgO nanoparticles. In this work, nanoparticles with different sizes were labeled as M11, M42, and M86 according to the mean particle size of the samples.

Two system nanofluids were prepared, which were composed of CTAB in solution and dispersed MgO nanoparticles. It is worth mentioning, this nanofluid was prepared by adding nanoparticles and CTAB at the same time. In this process, there is a competition between the surfactant–surfactant and surfactant–nanoparticles interaction.

Meanwhile, the system II nanofluids were composed of MgO nanoparticles with CTAB adsorbed on their surface (nanocomposite) based on the interaction surfactant–nanoparticles obtained from the adsorption isotherms, which varied the initial concentration of CTAB by 330, 660, and 1000 mg·L^−1^ for each test.

#### 2.2.2. Nanoparticles Characterization

The hydrodynamic diameter of the nanoparticles was measured by dynamic light scattering (DLS) employing a Nanoplus 3 from Micromeritics (Norcross, GA, USA) using a 0.9 mL glass cell at 25 °C. The sample was previously sonicated in deionized water for four hours at room temperature. The DLS technique measures the translational or rotational diffusion coefficients at a series of scattering angles [39]. This relation is expressed by the Stokes–Einstein equation in a dilute dispersion, as follows [39]:

(1) $$D_{p}=\frac{K_{B}T}{3\pi \eta D_{a}}$$

where, K_B_ is the Boltzmann’s constant (1.38 × 10^−23^ J·K^−1^), *T* (K) is the absolute temperature, *η* (cP) is the viscosity of the suspending medium, and *D*_a_ (m^2^∙s^−1^) is the diffusion coefficient of the nanoparticles. DLS measurements were performed by triplicate to ensure the repeatability of the test, obtaining uncertainties of ±0.1nm.

Additionally, the morphology of the MgO nanoparticles was characterized through high-resolution transmission electron microscopy (HR-TEM) using a Tecnai G2 F20 microscope (FEI, Hillsboro, OR, USA) and the crystalline structure was characterized by X-ray diffraction (XRD) using X’Per PRO (PANalytical) X-ray diffractometer with Cu Kα radiation λ = 1.5406 Å, at the scanning rate of 0.050.

Besides, the zeta potential (ζ) of the three synthesized nanoparticles was measured as a function of the solution pH using a Nanoplus 3 from Micromeritics (Norcross, GA, USA). The samples were previously sonicated for 3 h at 25 °C and added to aqueous solutions with pH = 2 and pH = 10, prepared with aliquots of sodium hydroxide 0.1 M and hydrochloric acid 0.1 M to obtain basic and acidic pH values that were measured using a Horiba D-54 pH meter (Horiba Instruments Inc., Kyoto, Japan). The point of zero charge (pH_pzc_) obtained at a pH value was ζ = 0. Zeta potential measurements were performed by triplicate to ensure the repeatability of the process, obtaining uncertainties of ±0.01 mV.

Fourier transforms infrared (FTIR) spectroscopy was performed using an IRAffinity-1 FTIR device (Shimadzu, Kyoto, Japan), which has a Michelson interferometer with an angle of incidence 30°. KBr pellets were macerated with the nanoparticles for the preparation of the sample in a 30:1 ratio by weight. For measurements, 5 mg of the mixtures were placed in the sample holder, and the test was carried out in the range of 400–4000 cm^−1^ using a KCl cell with 0.25 mm spacing at room temperature.

#### 2.2.3. Adsorption and Desorption Tests of CTAB onto MgO Nanoparticles

The adsorption and desorption experiments were performed to analyze the interaction between the surfactant and the nanoparticles. The adsorption isotherms between the surfactant (CTAB) and MgO nanoparticles were carried out in a Genesys 10S UV-Vis spectrophotometer (Thermo Scientific, Waltham, MA, USA) with a ±0.001 a.u. of uncertainty in the absorbance measurement. The sorption experiments were carried out at three different temperatures, 25, 35, and 45 °C, and the supernatant was measured at a fixed wavelength of 296 nm. The adsorption measurements were corroborated through thermogravimetric analyses, as reported in previous works [40,41,42]. Surfactant concentrations ranged between 1000 and 10,000 mg·L^−1^, and the nanoparticles’ dosage in the solution volume was fixed in 1200 mg·L^−1^. For the adsorption isotherms, the solutions were stirred for 2 h and left to stand for 24 h to ensure the adsorption of the surfactant molecules onto the surface of the nanoparticles. The adsorbed amount (*N*_ads_) in units of mg of surfactant per g of nanoparticles was determined according to Equation (2)

(2) $$N_{ads}=\frac{C_{o}-C_{E}}{m}V$$

where, *C_0_* (mg·L^−1^) is the initial concentration, *C_E_*(mg·L^−1^) is the equilibrium concentration, *V* (L) is the volume of the solution, *m* (g) is the dose of nanoparticles added to the solution, and *N*_ads_ (mg·g^−1^) is the amount of surfactant adsorbed onto the nanoparticle surface.

The desorption test was performed using batch-mode experiments [43] to evaluate the magnitude of the interaction forces between CTAB and the nanoparticles after adsorption. In the desorption experiments, MgO nanoparticles with CTAB adsorbed on their surface were placed in deionized water solution under constant stirring for 2 h and left to stand for 24 h. After that, the released surfactant in the solution was measured, and the remaining amount adsorbed *N*_ads,rem_ (mg·g^−1^) was calculated as follows:

(3) $$N_{ads,rem}=N_{ads}-\frac{C_{E,rem}}{m}V$$

where, *C_E,rem_* (mg·L^−1^) is the surfactant concentration in the solution after desorption. The desorption percentage was calculated as (Equation (4))

(4) $${\%}_{des}=\frac{M_{des}}{M_{ads}}⊗100$$

where, *M_ads_* (mg) is the adsorbed mass of surfactant before the desorption process, and *M_des_* (mg) is the desorbed mass of surfactant from nanoparticles.

#### 2.2.4. Fines Migration Tests in Sand Packs

**Figure 1:**
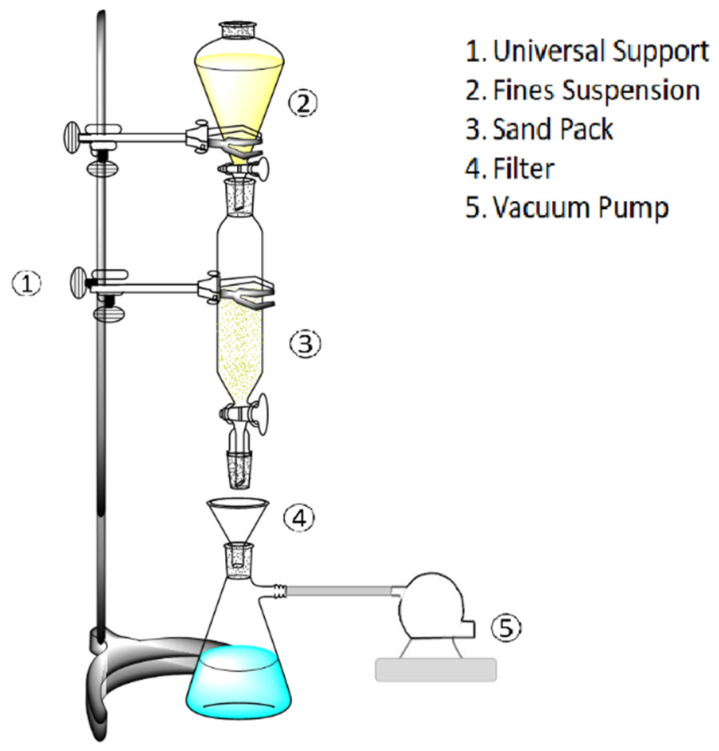
Experimental set-up for the graphical representation of fines migration laboratory tests in sand packs at atmospheric conditions (0.1 MPa and 25 °C).

The simulation of the porous media was performed using 20–40 US mesh Ottawa sand packed beds. Initially, the sand used was washed several times to eliminate any dirtiness. After, the sand was filtrated and dried at 120 °C for 4 h. Different treatments were employed including MgO nanoparticles dispersed in deionized water, CTAB solutions, nanofluids composed by the mix of MgO nanoparticles and CTAB, and the obtained nanocomposite (MgO nanoparticles with CTAB adsorbed) dispersed in deionized water. For this, 70 g of the sand were submitted to an impregnation process with each treatment for 24 h and further dried for 8 h at 80 °C. Figure 1 shows the setup employed for the fines retention tests. The fines suspension in all experiments was fixed in 2000 mg∙L^−1^ of kaolinite, considering this kind of fines as the most problematic due to their chemical instability and the average amount present in the migrating fines in Colombian field cases [35].

Initially, the retention capacity of the packed beds was compared varying the size of the nanoparticles used. Then, the nanoparticles with the best performance were selected and used in the following treatment evaluation at different concentrations between 1000 and 2000 mg·L^−1^. The influence of CTAB in the absence of the nanoparticles was also evaluated. Finally, the synergy of CTAB and MgO nanoparticles was evaluated in two systems. The system I refers to nanofluids composed of CTAB in solution and dispersed MgO nanoparticles; and system II nanofluids composed of MgO nanoparticles with CTAB adsorbed on their surface (nanocomposite), varying the initial concentration of CTAB by 330, 660, and 1000 mg·L^−1^ for each test.

#### 2.2.5. Design of Experiments

To find the optimal concentration for the treatment in the system I and then compare it with the variation of the concentration in the nanocomposite adsorbed component for the system II, a simplex-centroid mixture design (SCMD) was developed using the STATGRAPHICS Centurion software (XVI version, StatPoint Technologies Inc., Addison, TX, USA). A three-component SCMD was used considering the combination of different treatments, containing deionized water as the carrier fluid, magnesium oxide nanoparticles and hexadecyltrimethylammonium bromide (CTAB) in different proportions, to predict the effects in the retention capacity of the porous media. The proportion of each component in the mentioned experiments must satisfy the following restriction (Equation (5)) [44]:

(5) $$\sum_{i}^{q}x_{i}=x_{1}+x_{2}+x_{3}+\cdots +x_{q}=1,\;x_{i}\geq 0$$

where, *q* refers to the number of components varying in the mixture. Then, for the current work, *q* = 3, corresponding to the three components expressed before. The selected range of concentrations for each component consists of:

0.98 ≤ *Water* ≤ 1 (6)

0.02 ≤ *MgO nanoparticles* ≤ 0 (7)

0.02 ≤ CTAB ≤ 0(8)

The simplex−centroid mixture design considers another parameter *m* that is related to the order of the statistical model used for the design [45]. The rule to select the concentrations of the treatment consists of the possible combination of the proportions presented below, in Equation (9):

(9) xi=0, 1m, 2m, ⋯ mm

**Figure 2:**
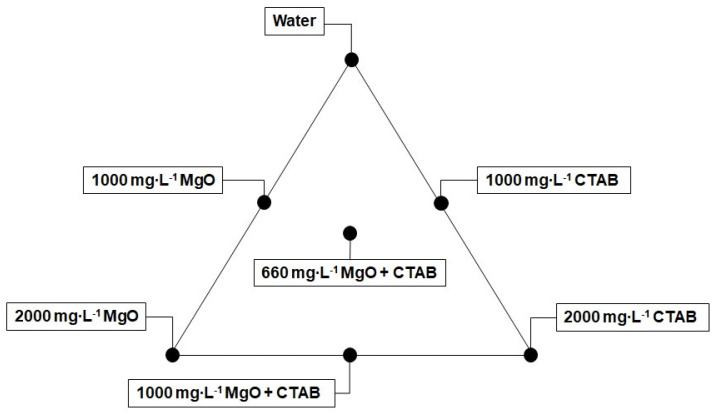
Three components simplex−centroid mixture design proposed to evaluate the synergistic effect between MgO and hexadecyltrimethylammonium bromide (CTAB) in the system I with different mass fractions up to 2% using deionized water as the carrier fluid.

Specifically, for a cubic model, the rule for the experiments indicates that maximum and minimum concentration of each compound must be evaluated, as well as other points in which the three components of the mixture are related. In this sense, looking for a treatment that maximizes the capacity of the medium to inhibit the fines migration, there were evaluated seven points considering the restrictions mentioned before as shown in Figure 2.

The regression model for the variable of response, which is related to the retention capacity of the medium, was established using a special cubic regression fitting. The regression model equation is:

(10) $$y=\beta_{1}x_{1}+\beta_{2}x_{2}+\beta_{3}x_{3}+\beta_{12}x_{1}x_{2}+\beta_{13}x_{1}x_{3}+\beta_{23}x_{2}x_{3}+\beta_{123}x_{1}x_{2}x_{3}$$

where, *y* is the variable response, *x_i_* are the fractions of each component and *β_i_* are the coefficients of the linear terms, *β_ij_* are the components of the binary mixtures, and *β_ijk_* are the coefficients of the ternary mixture. In this study, *x*_1_ = water, *x*_2_ = MgO nanoparticles, and *x*_3_ = CTAB.

#### 2.2.6. Core Flooding Test

**Figure 3:**
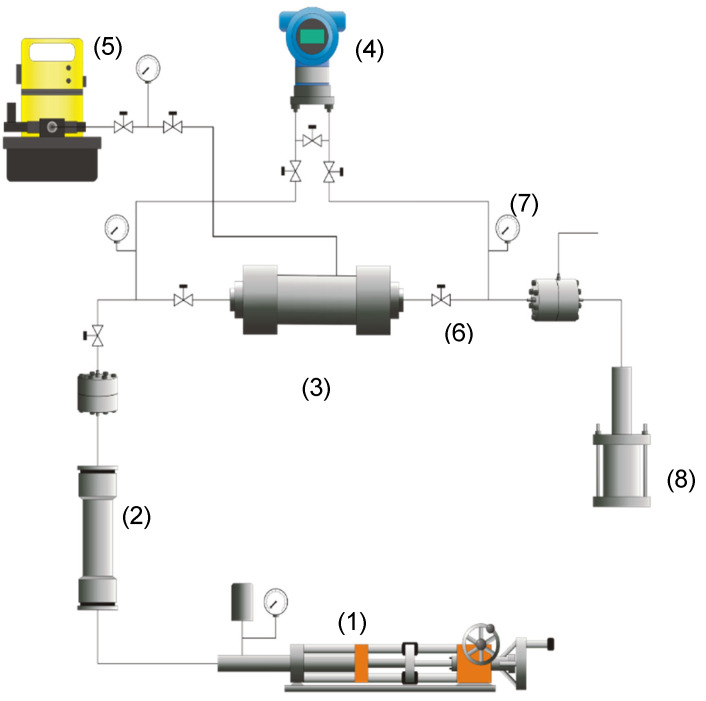
Schematic representation of the experimental setup for the displacement tests: (**1**) displacement pump, (**2**) cylinder, (**3**) sample core holder, (**4**) pressure transducer, (**5**) hydraulic pump, (**6**) valves, (**7**) manometers, and (**8**) a pressure multiplier.

The core flooding experiment was performed to evaluate fines migration inhibition at reservoir conditions considering the impact of the optimized MgO nanoparticles treatment with CTAB based on the critical rate evaluation. The experimental setup consists of one displacement pump (DB Robinson Group, Edmonton, AB, Canada), a pressure transducer (Rosemount, Emerson, Chesterfield, MO, USA), one cylinder (Max Servicios, S.A.S., Medellín, Colombia) containing synthetic brine, manometers (Rosemount, Emerson, Chesterfield, MO, USA), fraction collectors, a hydraulic pump (Enercap, Actuant Corporation, Milwaukee, WI, USA), and a pressure multiplier. The experimental arrangement is shown in Figure 3 and Table S1 of the supplementary material summarizes the properties of the porous medium used.

First, measurements of the absolute permeability (*K*) and the effective permeabilities of water (*K*_w_) and oil (*K*_o_) were performed. For this, the fluids were injected in the order water–oil-water at a rate of 2 mL·min^−1^ until residual saturation of each phase was achieved. The critical rate was determined with the injection of water at different flow rates increasing in intervals of 0.3 mL·min^−1^ until detecting a decrease of at least 10% in *K*_w_. Then, water was injected in an opposite flow of the production direction at critical flow to recover the permeability of the system and then was injected in production direction to ensure that the permeability was restored. The same procedure was developed for oil injection, to obtain the critical rate in which *K*_o_ decreases by at least 10%. The permeability of the system was restored. Then, 0.5 pore volumes (PV) of the optimized nanofluid were injected at a 0.2 mL∙min^−1^ and the porous medium was left to soak for 12 h. The critical rate was measured following the protocol previously described. Overburden pressure was fixed at 34.5 MPa, pore pressure at 6.9 MPa, and system temperature at 93 °C.

## 3. Modeling

### 3.1. Adsorption Model

The solid–liquid equilibrium (SLE) model was used to describe the adsorption isotherm of CTAB onto the nanoparticles. It consists of a model developed by Montoya et al. [46] related to the adsorption of self-associative molecules like surfactants on the surface of the nanoparticles from different natures [40,41,47]. The model describes the concentration of the surfactant in equilibrium *C* (mg∙L^−1^) as follows:

(11) $$C=\frac{\psi H}{1+K\psi }e^{\left(\frac{\psi }{N_{m}}\right)}$$

*H* (mg·g^−1^) is related to the Henry’s Law constant, *K* (g·g^−1^) is an indicator of the association of surfactant molecules once the primary sites are filled, and *N*_m_ (g·g^−1^) parameter refers to the maximum adsorption capacity:

(12) $$K=\frac{K_{T}RT}{SA}$$

(13) $$\psi =\frac{-1+\sqrt{1+4K\xi }}{2K}$$

where, *K_T_* is the reaction constant of the dimer formation, *SA* is the adsorbent surface area, and ξ is a constant described in Equation (14) considering that *N* (g·g^−1^) is the amount of adsorbed surfactant:

(14) $$\xi =\frac{N_{m}N}{\left(N_{m}-N\right)}$$

### 3.2. Breakthrough Curves

The modeling of the retention dynamics on the packed beds was based on the models developed by Clark [48] and Wolborska [49]. The Clark model is based on the concept of mass transfer as follows [48]:

(15) $${\left(\frac{C}{C_{o}}\right)}^{n-1}-1=Ae^{-rt}$$

where, *C* (mg·L^−1^) is the initial effluent concentration of the solution at the outlet, *C_o_* (mg·L^−1^) is the influent concentration of the fines solution at the inlet of the bed, A and r are Clark constants specific for each material according to their kinetic properties, and *t* (min) is the time taken until the saturation of the medium. The relation of the Clark constants is presented below:

(16) $$A=e^{\left(\frac{rN_{o}Z}{C_{o}U}\right)}$$

where, *Z* (cm) corresponds to the sand packed bed height, *U* (cm·min^−1^) is the linear velocity of injection of the fines solution, and *N_o_* (mg·L^−1^) corresponds to the exchange capacity of the medium. Additionally, for predicting the lower concentrations of the breakthrough curves the Wolborska model was used [49]. It consists in a relation of different mass transfer equations [49], as follows:

(17) $$\ln\left(\frac{C}{C_{0}}\right)=\frac{\beta C_{0}}{N_{0}}t-\frac{\beta }{U}Z$$

where the constants presented in both models represent the same variables, also there is introduced another parameter *β* (min^−1^) that represents the kinetic coefficient of the external mass transfer.

## 4. Results

### 4.1. Nanoparticle Characterization

**Figure 4:**
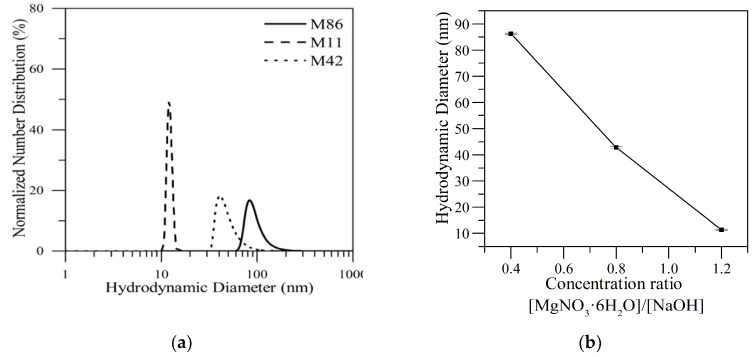
(**a**) Normalized number distribution of three different sizes of MgO nanoparticles 11 (M11), 42 (M42), and 86 nm (M86) based on DLS measurements at 25 °C, where the number of the nomenclature refers to the hydrodynamic diameter (nm) found for each particle, and (**b**) the size of MgO nanoparticles related to the variation of the concentration ratio of the precursor.

Initially, the size of the three different synthesized nanoparticles was measured. Figure 4 shows the normalized number distribution of the synthesized nanoparticles at 25 °C. The samples are named according to the mean size (nm) of each particle find through DLS tests, meaning the M11 nanoparticles refer to the MgO nanoparticles with 11 nm of hydrodynamic diameter. The relationship between the concentration of the precursor and the size obtained by DLS is inversely proportional. That phenomenon is presented because, during the growth processes, the excess of NaOH favors the aggregation of particles [37]. Considering that nucleation rates will be highest for substances with low solubility under a very high pH compared to a low pH, it is presented faster primary nucleation caused by the reduction of the solubility of Mg(OH)_2_ [50]. The nuclei formed during the process are immediately negatively charged with OH^−^ ions adsorbed due to strongly basic conditions. After, these interconnected with each other via hydrogen bonds, which gave rise to agglomerations [51].

Figure S1 of the supporting material shows the TEM micrographs for three samples evaluated, where the average particle size observed of 86, 48, and 11 nm is related to the different ratios of magnesium nitrate hexahydrate/sodium hydroxide (MgNO_3_·6H_2_O/NaOH) used in the nanoparticles synthesis, with values of 0.4, 0.8, and 1.2 respectively. Besides, the XRD analysis confirms the formation of MgO nanoparticles. The peaks at 2*θ* values of 36.9°, 42.9°, 62.3°, and 74.6° and 78.6° in the 2*θ* range can be indexed to the (1 1 1), (2 0 0), (2 2 0), (3 1 1), and (2 2 2) planes of the face-centered cubic (FCC) structured MgO nanoparticles. No other peaks were detected in the XRD pattern confirming the high purity of the synthesized materials.

Figure S2 shows the zeta potential of nanoparticles’ dispersion in aqueous solution as a function of pH at 25 °C. The measurement of the zeta potential varying the pH of the sample indicates that the point of zero charge (pH_pzc_) is between 11 and 12 for the three synthesized nanoparticles, which is in agreement with the values reported in the literature. There are small differences in the value of the point of zero charge between the three samples of nanoparticles. The (pH_pzc_) results are very important for the application of fines retention because the MgO nanoparticles alter the zeta potential of the porous medium, turning the rock surface into a positive one, and allowing the attraction of fine particles with negative charges despite the alkalinity of the medium [25,52]. Table 1 summarizes the results of the characterization for the three samples of nanoparticles related to the average size found for the particles.

**Figure 5:**
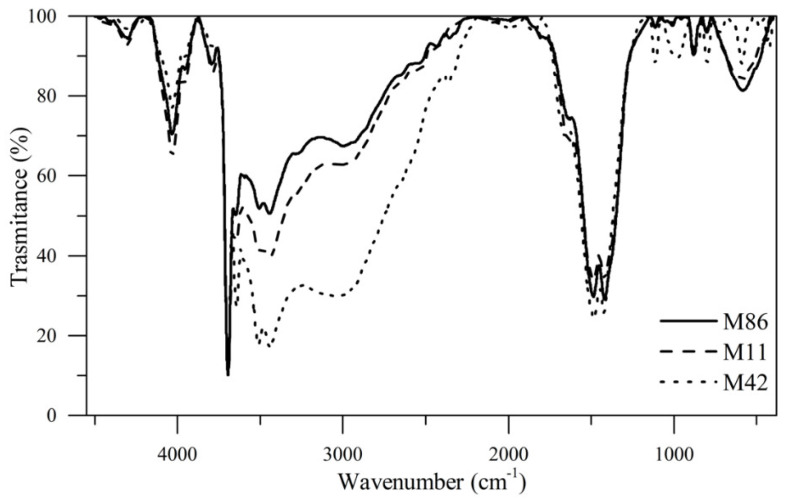
FTIR spectra of the three different sizes of MgO nanoparticles 11 (M11), 42 (M42), and 86 nm (M86) at 25 °C.

The Fourier transform infrared spectrum (FTIR) of the MgO nanoparticles is shown in Figure 5. The bands observed at 3439 and 4020 cm^−1^ correspond to the OH stretching mode of the hydroxyl groups present on the surface due to the humidity. Moreover, the band at 1485 cm^−1^ was attributed to the bending vibration of the water molecule. Metal oxides generally show bands in the fingerprint region below 1000 cm^−1^. The bands observed in FTIR spectra at 880, 545, and 580 cm^−1^ suggest the presence of Mg–O stretching vibrations, which is attributed to the vibrations of metal-oxygen bonds in the synthesized material [53]. The reduction of that band for the M86 sample is possibly related to the significant presence of OH^−^ due to the excess of NaOH used in the synthesis reaction. The band at 3700 cm^−1^ is due to the O–H vibration of the brucite phase of Mg(OH)_2_, namely, –OH stretching vibration bonded with Mg, while bonding vibration with Mg and a bending bond is presented at 1415 cm^−1^ [54]. In this case, the intensity of the band at 370 cm^−1^, is similar in the analyzed samples due to the presence of the Mg-O functional group. The results are similar to those reported in the scientific literature [53,55,56,57].

### 4.2. Analysis of CTAB-MgO Nanoparticles Interaction

**Figure 6:**
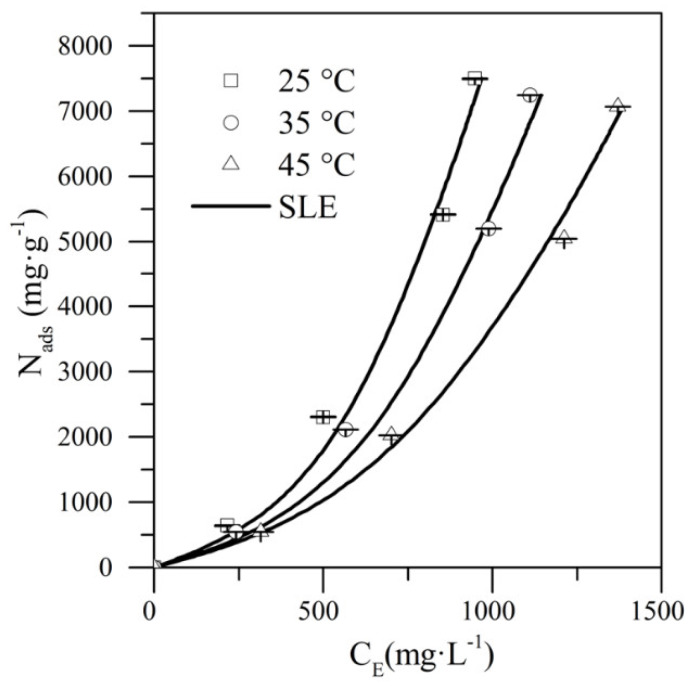
Adsorption isotherms for CTAB onto M11 nanoparticles at a fixed concentration of nanoparticles (1200 mg·L^−1^) varying the CTAB dosage below its critical micelle concentration (CMC) at 25, 35, and 45 °C using the solid–liquid equilibrium (SLE) model. *C*_E_ (mg·L^−1^) is the asphaltene equilibrium concentration and *N*_ads_ (mg·g^−1^) is the adsorbed amount.

To analyze the interaction between CTAB and MgO nanoparticles in its nanocomposite form of system II, there were made adsorption and desorption tests between these two components. Additionally, to evaluate the temperature effect in the adsorption phenomena of CTAB onto the surface of the M11 nanoparticles, the tests were developed at three different temperatures of 25, 35, and 45 °C and were described using the SLE model, which are shown in Figure 6.

Figure 6 shown the adsorption isotherms of CTAB on the MgO nanoparticles, which are according to the IUPAC classification type III isotherm [58]. This type of isotherm is characterized by the multilayer adsorption of the adsorbate (CTAB) on the adsorbent (MgO nanoparticles) surface [22], that is, once the first CTAB monolayer is formed on the MgO nanoparticles, this serves as active sites for the adsorption of more CTAB molecules, generating multilayer adsorption [59]. As observed in Henry’s region (*C*_E_ < 100 mg·L^−1^) associated with the affinity between the surfactant and the solid’s surface, there is a low affinity between the CTAB and the MgO nanoparticles.

Additionally, as shown in Figure 6, at a fixed value of equilibrium concentration, a smaller amount of adsorbed surfactant is obtained by increasing the temperature. This result indicates a negative influence of temperature in the attractive forces between the couple MgO–CTAB. This behavior is also related to a change in the aggregation state of CTAB surfactant, considering that it could be adsorbed as micelles or individual molecules [40]. Using the Five-parameter SLE model [46], a thermodynamic study of the adsorption phenomena was developed. Changes in entropy $\Delta S_{\mathrm{ads}}^{0}$, enthalpy $\Delta H_{\mathrm{ads}}^{0}$, and Gibbs free energy $\Delta G_{\mathrm{ads}}^{0}$ were calculated. As adsorption is exothermic, $\Delta H_{\mathrm{ads}}^{0}$ was negative and estimated equal to −0.0988 kJ·mol^−1^. Besides, $\Delta S_{\mathrm{ads}}^{0}$ was 0.00033 J·(mol·K)^−1^, confirming the increment of the randomness and disorder of the system at the liquid–solid interface [40]. The positive value of $\Delta S_{\mathrm{ads}}^{0}$ also corresponds to an increase in the degree of freedom of the adsorbed species. This causes the CTAB molecules to remain in the solid phase instead of the liquid phase, indicating a high affinity for nanoparticles [60]. This result is in agreement with Helgeson et al. [61], who established that CTAB uptake by silica nanoparticles is characterized by positive values of $\Delta S_{\mathrm{ads}}^{0}$ and negative values of $\Delta H_{\mathrm{ads}}^{0}$. Besides, several reports of adsorption of surfactants and other amphiphilic molecules like asphaltenes over solid surfaces (i.e., nanoparticles) have shown the same behavior [40,46,62]. Finally, the feasibility of adsorption process is clarified by $\Delta G_{\mathrm{ads}}^{0}$, estimated in −0.189 J·mol^−1^, −0.192 J·mol^−1^, and −0.195 J·mol^−1^ at 25, 35, and 45 °C, respectively, corroborating the spontaneity of the adsorption processes.

**Figure 7:**
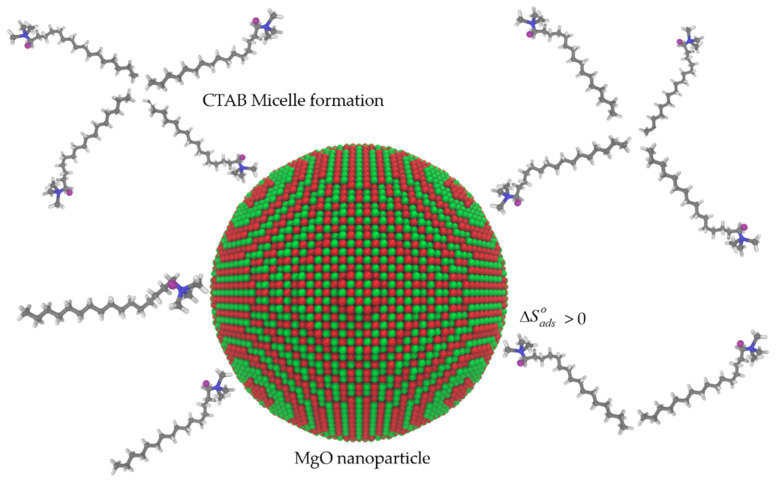
Conceptual scheme of the interaction between the MgO nanoparticles and CTAB surfactant molecules for the nanocomposites studied in System II based on the energy minimization for each of the components using molecular dynamics. Magnesium is shown in green, oxygen in red, carbon in grey, hydrogen in white, and bromium in purple.

Figure 7 shows a conceptual scheme of the CTAB–nanoparticles interaction based on energy minimization for CTAB and nanoparticles by molecular dynamics using the VDM visualization software to obtain images [63].

As seen in Figure 7, the interaction of the surfactant adhered onto the nanoparticle surface in system II favors the attractive forces between the migrating fines and the porous media. It is important to mention that this scheme can be a representation of system II where the CTAB concentrations for the three evaluated tests were under the critical micelle concentration (CMC) that occurs at surfactant concentrations close to 4000 mg·L^−1^ [40]. Meanwhile, system I is different to the one shown in system II, where the nanoparticles and the surfactant are added simultaneously to the water, leading to a possible competition between the surfactant molecules attraction and the interaction of the surfactant with the nanoparticles or the rock grains [40].

Regarding the SLE parameters showed in Table 2, the *H* values are increased with the increase of the temperature. It shows that the adsorption affinity decreases with a rise in the temperature in the system as this parameter is an indicator of the preference of the surfactant for being in the liquid phase rather than being adsorbed on a solid surface. That argument is verified with the inverse relation between *N*_m_ values and temperature changes. The *K* value increases with a temperature increase, indicating that the adsorption at lower temperatures leads to a higher self-association of the CTAB on the MgO nanoparticles surface.

**Figure 8:**
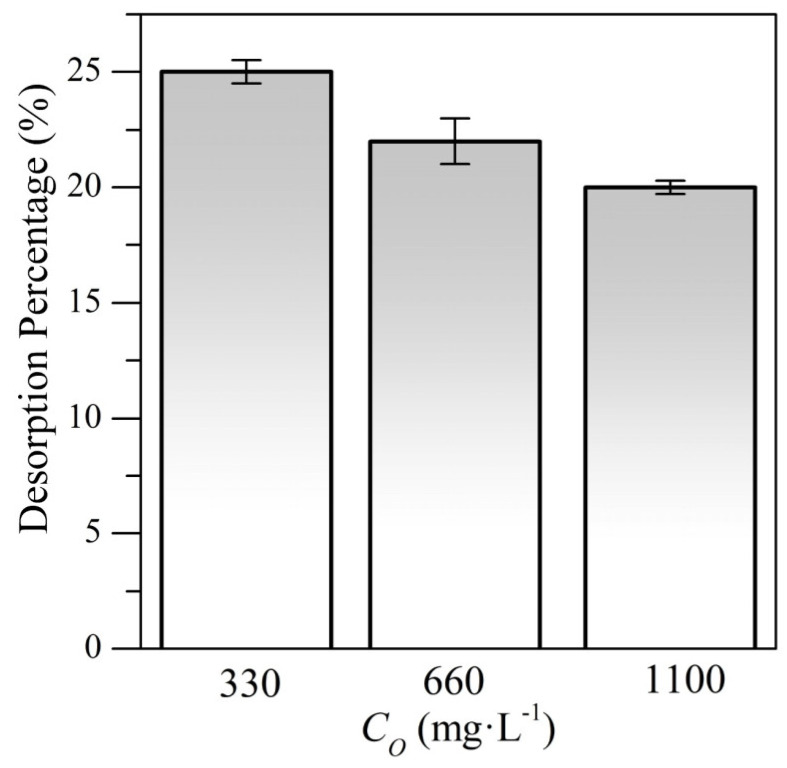
Percentages of desorption for different initial concentrations (*C*_0_) of CTAB and a fixed concentration of MgO nanoparticles (1100 mg·L^−1^) for system II at 25 °C.

Figure 8 shows the percentage of desorbed CTAB after 24 h at 25 °C. The different CTAB concentrations were chosen to analyze the performance of the nanocomposites in comparison with treatment in the system I, these tests allow the estimation of the real concentration of CTAB onto the surface of the nanocomposite. The nanocomposite mixtures were named according to the amount of CTAB portion adsorbed onto the surface of the nanoparticles in units of mg∙L^-1^ such as 247CTAB, 515CTAB, and 800CTAB, referring to 247, 515, and 800 mg∙L^−1^ respectively.

As Figure 8 shows, a slight reduction in the value of percentage desorbed with the increase of CTAB concentration was observed. This could indicate that a higher CTAB concentration would allow stronger interaction between the components, hindering the desorption to the aqueous phase. These results showed that CTAB-MgO nanoparticles interactions were strong enough to avoid surfactant desorption percentages higher to 25%, which is the maximum desorption percentage obtained at 330 mg·L^−1^ of CTAB. Finally, it is important to mention that free CTAB in the suspension of the nanocomposites helps the colloidal stability of the nanoparticles, allowing these to stay in suspension and avoiding aggregation. Making a comparison between the amount of free CTAB in each system, this is higher for Systems I than Systems II in all cases.

### 4.3. Effect of Individual Components in the Inhibition of Fines Migration

#### 4.3.1. Effect of CTAB

**Figure 9:**
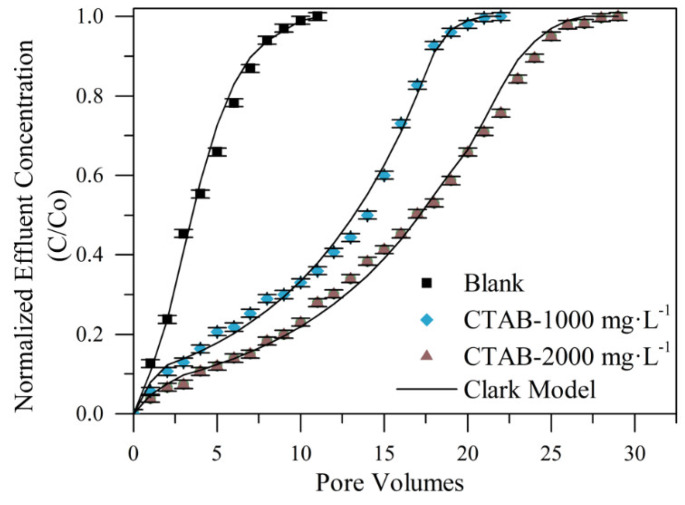
Breakthrough curves for two different concentrations of the CTAB treatment of system I in Ottawa 20/40 sand packed bed assessed with kaolinite fines at 0.2% in mass fraction solution and 25 °C.

CTAB surfactant as a treatment for fines particle retention was evaluated. For this, different CTAB dosages were used (1000 and 2000 mg·L^−1^). Therefore, the first fluid evaluated was composed of a CTAB-containing aqueous solution in the absence of nanoparticles. Figure 9 shows the breakthrough curves to visualize the trapping effect on the kaolinite particles flowing through the packed bed with the use of the CTAB concentrations of 1000 and 2000 mg·L^−1^, together with the Clark model fitting. From Figure 9, low retention of the particles was observed when the bed was not impregnated with the CTAB-containing aqueous solution (Blank). The effluent obtained from the first porous volume injected (PVI) has suspended kaolinite particles, and the breaking point of this curve was observed from the second PVI. On the other hand, by treating the bed with the CTAB, there was higher retention of the migratory particles within the sand column, regardless of the concentration. At the beginning of these curves, it was observed that the effluent was practically free of fines until the third porous volume where the breakpoint was located. Furthermore, the saturation of the bed occurred between 18 and 25 PVI, while the untreated system saturated after the eight PVI. The good performance of CTAB-based treatment is due to the amine-based cationic surfactants that can attract negatively charged components such as clay fines [19]. Most of the amine-based clay stabilizers commonly used work on the principle of substitution of cationic species in the clay lattice [14].

The increase in the stabilizing property of the porous medium after the treatment with the CTAB was observed by comparing the system with the untreated packed bed, which shows that the application of the cationic surfactant treatment efficiently increased the retention of fines by 200% in the CTAB-1000 mg·L^−1^ treatment and 264% in the CTAB-2000 mg·L^−1^. This is explained by the faculty of the cationic species to stabilize fine clay particles [14,19,20]. Comparing both concentrations evaluated, the increase in the CTAB concentration allowed a higher number of fines retention by the porous medium before saturation. Nevertheless, with the increase in CTAB concentration, the increase in the fines trapped was not enough higher, displacing the breakpoint by three pore volumes.

#### 4.3.2. Effect of Nanoparticles

To analyze the effect of the nanoparticles size and the variation of nanoparticles concentration on the inhibition of fines migration, the three different sizes of MgO nanoparticles 11 (M11), 42 (M42), and 86 nm (M86) at a fixed concentration were evaluated. The use of these nanoparticles to control the fines migration in the beds demonstrated good results for all sizes and concentrations, compared with the untreated sand packed bed. From the breakthrough curves obtained for each system, it was observed that for the same number of PVI, the system treated with the nanoparticles always had a smaller amount of fines in the effluent compared to the virgin bed (Blank). The good behavior of these systems could be associated with the pH_PZC_ value, which was close to 12 for the three nanoparticles, regardless of its hydrodynamic diameter, indicating that at the pH of the fines solution the nanoparticles had positive surface charges. Thus, attractive forces are dominant by Van der Waals and electrostatic forces [8,18,23,64].

**Figure 10:**
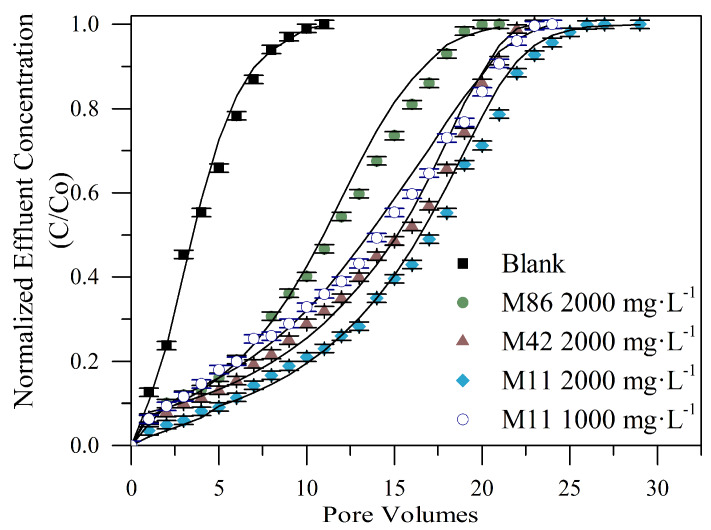
Breakthrough curves for the treatments of three different MgO nanoparticles M11 (at 1000 and 2000 mg·L^−1^), M42 (at 2000 mg·L^−1^), and M86 (at 2000 mg·L^−1^) of the system I in Ottawa 20/40 sand packed bed assessed with a kaolinite 0.2% in mass fraction solution at 25 °C.

However, different yields were obtained as the size of the nanoparticles varied. It should be noted that this variable was not reported before in other studies related to the effect of fines migration inhibition treatments. Figure 10 shows the breakthrough curves corresponding to the treated beds with M11, M42, and M86 nanoparticles at a fixed concentration of 2000 mg·L^−1^, and M11 nanoparticle also evaluated at a concentration of 1000 mg·L^−1^ as it showed the best performance.

As it can be observed from Figure 10, the blank bed reached the saturation point in the 8th porous volume injected, which shows that the application of nanofluids efficiently increased the retention of fines in the porous medium by 245%, 209%, and 190% using M11, M42, and M86 respectively. In the case of the M11 nanoparticles assessed at 1000 mg∙L^−1^, the increase was about 218%. The performance of the blank bed represents the porous medium at the reservoir where the detachment of the fines is produced by the effect of the double layer and the reduction of van der Waals attraction forces that allow the repulsive forces to become more significant [6,9].

Figure 10 shows an inversely proportional ratio between the nanoparticle size and the increase in the retention capacity of the medium to inhibit the fines migration. This result can be related to the different surface area of the materials (see Table 1) [65], showing the highest retention for the smallest material, i.e., the one with the highest surface area allows higher surface reactivity and a higher amount of available functional groups [66]. Therefore, the smallest nanoparticles represent the best alternative of the synthesized treatment. Figure 10 also shows that the difference in adsorbed amount was higher between M86 and M42 than the difference between M42 and M11, this indicates that for field applications the selection of M42 and M11 nanoparticles could be made based on the porous medium conditions rather than the retentive capacity of the kaolinite fines such as is presented by Franco et al. [24] in the different nanotechnology field applications to enhance oil and gas productivity and recovery.

About the analysis of the variation in the M11 nanoparticles concentration, the results showed an increase in the inhibition capacity related as the concentration increased. For the evaluated concentrations a high quantity of the nanoparticles allowed more interaction with the displaced solids and also favored the attractive electrostatic forces between unattached fines and the porous matrix; consequently, a higher retention capacity was reached, which was in agreement with previous evaluations that have shown that the increase of nanoparticles’ concentration leads to higher efficiency in the fixing of the fines migration problem [36,52].

However, a slight difference in the retention capacity between both concentrations of M11 nanoparticle was observed, where the increase of the nanoparticle’s concentration to 2000 mg∙L^−1^ represents a 27% increase in the retention capacity of the medium. This suggests the importance to look for optimal dosage of nanoparticles that allows the system to achieve the benefit sought with the least concentration possible. Additionally, to take into account that the excess of nanoparticles could reduce the active sites with the nanoparticle–nanoparticle interactions instead of the nanoparticle–fine particle interaction. This is supported by Guzmán et al. [67], which have shown the importance of the nanoparticle dosage in a similar performance with nanotechnology applied in asphaltene-related treatments. Besides, for field applications, this optimal concentration could show more benefits in terms of the minimum concentration used and the high retention achieved comparing with other possible treatments.

### 4.4. Synergistic Effect between Nanoparticles and CTAB in the Inhibition of Fines Migration

#### 4.4.1. System I: Nanofluid with Free CTAB in Solution

**Figure 11:**
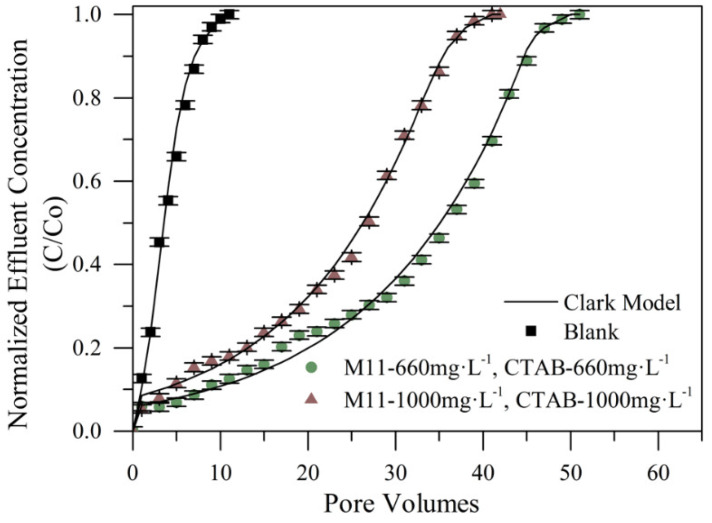
Breakthrough curves for different concentrations of MgO nanoparticles (M11)/CTAB of the system I in Ottawa 20/40 sand packed bed assessed with a kaolinite 0.2% in the mass fraction solution at 25 °C.

Nanofluids composed of CTAB and MgO nanoparticles at two different concentrations of 660 and 1000 mg·L^−1^ of each component, were prepared according to the SCMD presented below. In this case, the nanofluids consist of a nanoparticles’ suspension in a CTAB-based aqueous solution. For these evaluations, were selected the M11 nanoparticles as it shows the best performance in the previous tests. The obtained breakthrough curves are presented in Figure 11.

Figure 11 verifies the synergistic effect of mixing the components in the nanofluids. There were obtained increases in the inhibition of fines migration by 382% higher in the case of the maximum concentration evaluated (M11-1000 mg·L^−1^/CTAB-1000 mg·L^−1^ treatment), and a 464% increase in the retention capacity when the lower concentration was assessed (M11-600 mg·L^−1^/CTAB-600 mg·L^−1^ treatment), this represents a fines retention of about four times higher than the blank bed. There is a deposition of the surfactant in the surface of the nanoparticles in which the active sites of the porous medium and the surfactant micelles can interact, allowing an increase in the retention capacity of fines.

The results in Figure 11 indicate that the lowest concentration of free CTAB in the evaluated solution increased the active zones where the fines could interact with the porous medium. It is possible to attribute this phenomenon to the formation of CTAB micelles, because of the concentration of free-CTAB in the treatment [40]. The presence of these micelles could lead to a competition between the surfactant–rock interactions and the nanoparticle–rock interactions reducing the active sites and consequently the retention capacity of fine particles. In this sense, this phenomenon is more likely to occur at higher surfactant dosage (1000 mg·L^−1^ M11-1000 mg·L^−1^ CTAB). The results suggest that it should have a proper concentration of free CTAB for minimizing the formation of surfactant micelles and therefore increasing the retentive capacity of the porous medium, and this is why it is considered proper to use a design of experiments to find the optimal concentration of both compounds.

#### 4.4.2. Statistical Analysis for the DOE

Table 3 shows the estimated parameters of the Clark and Wolborska models to predict the performance of each treatment. The *β* parameter in the Wolborska model for the treatments evaluated indicates the kinetic coefficient of mass transfer. Then, the lower value was obtained for the blank because the packed bed could not trap a high amount of fines until saturation, so the mass transfer between the sand and the fines effluent was small. For the other treatments, at a lower concentration of the breakthrough curves, the kinetic parameter did not vary significantly due to similarity in the behavior of the curves. Even that, the parameter got higher values for the treatments with better performance. That indicates higher retention of solids in the sand at lower concentrations in the breakthrough curves.

Accordingly, the *N*_0_ parameter of the Clark model refers to the capacity of the medium to adsorb a specific amount of fines per volume unit [48]. Then, there is a tendency shown in the parameter and the number of volumes injected into the medium. So, the treatment with the highest capacity to absorb fines was the 660 mg·L^−1^ M11/CTAB solution, and it was in agreement with the experimental results. For the CTAB treatments, the parameter suggests that no representative enhancement in the treatment performance occurred with the increase in the CTAB concentration.

**Figure 12:**
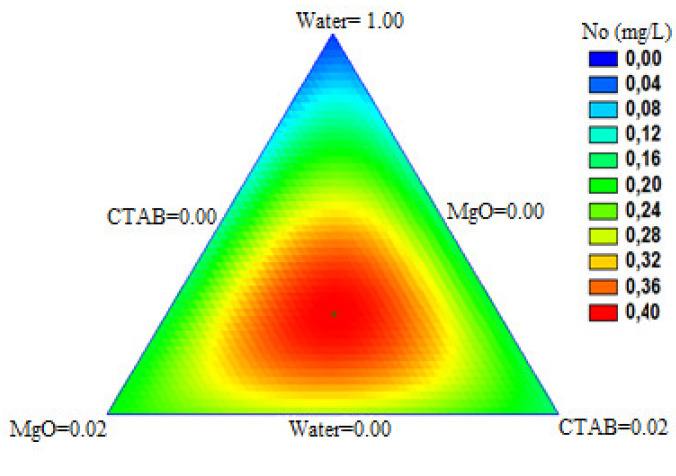
Response surface for the three compounds evaluated (MgO nanoparticles, CTAB, deionized water) obtaining the *N*_0_ (mg·L^−1^) parameter according to the cubic special model of the simplex-centroid mixtures design of experiments.

Hence, the *N*_0_ parameter was selected for the optimization of the nanoparticles and surfactant dosage. Figure 12 shows the dependence of the *N*_0_ parameter with the dosage of the components in the ternary mixture. It is worth mentioning that the maximum value that it could reach (*N*_0_ = 0.40 mg·L^−1^) was very close to the one found with the 660 mg·L^−1^ M11 /CTAB test that was *N*_0_ = 0.39 mg·L^−1^.

Figure 12 shows that the worst results were related to the treatments with lower nanoparticles dosage. On the other hand, the warm-colored sections that were situated in the central location of the image show similar patterns between the *N*_0_ values and the variation of both MgO nanoparticles or CTAB concentration. It is also evident that higher *N*_0_ values represented by red color area were reached in treatments that mix CTAB and MgO nanoparticles in similar proportions, consequently with the results found experimentally.

The results obtained from Figure 12 and the model proposed indicates that the *N*_0_ maximum value was 0.40 mg·L^−1^ and was reached at the concentration of 730 mg·L^−1^ MgO nanoparticles and 740 mg·L^−1^ of CTAB. Analyzing this result and comparing it with the *N*_0_ value of the 660 mg·L^−1^ M11/CTAB test (*N*_0_ = 0.39 mg·L^−1^) it was evident that there was not a considerable increase in the different concentration treatments. For this reason, it was selected the 660 mg·L^−1^ concentration of MgO nanoparticles and CTAB to evaluate and compare with the treatments in system II. The calculated parameters of the model are summarized in Table 4.

#### 4.4.3. System II: MgO Nanoparticles/CTAB Nanocomposite

**Figure 13:**
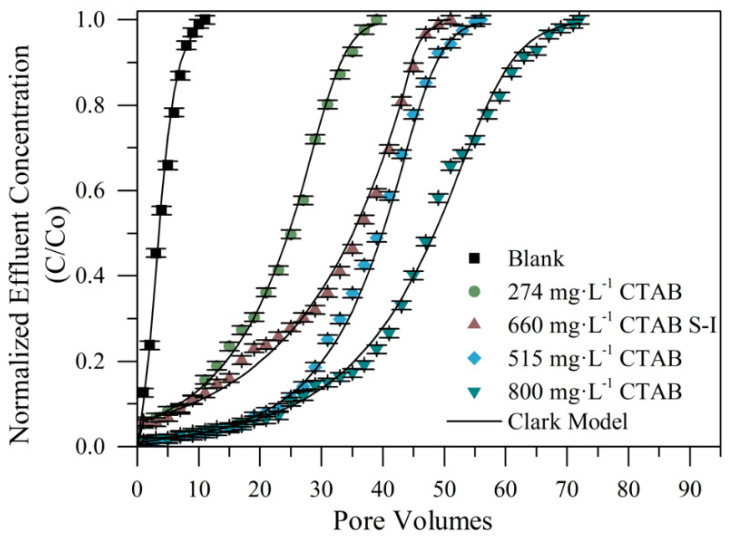
Breakthrough curves comparing results of free CTAB in system I (S-I) at a fixed concentration of nanoparticles (660 mg·L^−1^) with different percentages of CTAB adsorbed onto the nanoparticles at a fixed concentration of nanoparticles (660 mg·L^−1^) in system II, assessed with a kaolinite 0.2% in mass fraction solution at 25 °C.

To evaluate the effect of the nanocomposite on fines retention, three different retention tests were developed at different concentrations of CTAB adsorbed onto the nanoparticles. To study the effect of the surfactant adsorbed concentration, it was used a fixed MgO nanoparticles concentration (660 mg·L^−1^) that was found as the optimal MgO nanoparticles concentration based on the design of experiments presented in system I. Breakthrough curves obtained from the tests mentioned are shown in Figure 13, which are named as the real amount of CTAB portion adsorbed onto the nanoparticles’ surface in units of mg·L^−1,^ as 247CTAB, 515CTAB, and 800CTAB, respectively.

The synergistic effect of the CTAB adsorbed onto magnesium oxide nanoparticles surface was verified as shown in Figure 13, where it was obtained a fines retention higher of up to six times than that of the sand packed bed without any treatment in the best case, it represents an increase in the retention capacity of 654%. Additionally, comparing with the best result obtained from the combined compounds in solution (System I described in Section 4.4.1), the performance obtained of the treatment was about 200% higher than system I. Additionally, it has a trend of increasing the retention capacity as surfactant concentration increases onto the nanoparticle surface, favoring an adsorptive layer and minimizing the amount of free surfactant. It is worth remembering that in system II there are nanoparticles with CTAB adsorbed (nanocomposite) in the absence of free surfactant. The interaction of the nanocomposite in the aqueous system should be further studied as different phenomena such as aggregation, agglomeration, and colloidal bridging, among others, can occur.

Another important characteristic of the obtained breakthrough curves was the slope tendency of increment in the rupture point visible after the 10th, 25th, and 40th pore volume added, for the 247CTAB, 515CTAB, and 800CTAB treatments, respectively. It is an indicator of the higher magnitude of forces (like Van der Waals and double-layer repulsive force) and their affinity with the fines with an increase of the CTAB concentration used to functionalize the nanoparticles. There was an opposite case for the CTAB treatment suspended in solution (660 mg·L^−1^ CTAB system I), where the breakthrough point was ubicated immediately that began to pass the fines solution.

All the curves were modeled by Clark and Wolborska models. The answer parameters of those models for each treatment evaluated are present in Table 5.

The *N*_0_ parameter of the Clark model refers to the media capacity to adsorb a specific amount of fines per volume unit. Then, it shows an increase tendency respect to the CTAB concentration added to the specific treatment, verifying a better performance of the treatment with an increase of the surfactant concentration as is shown in Figure 12, therefore the best result was obtained to the breakthrough curve of 800 mg·L^−1^ of CTAB adsorbed.

The *β* parameter of the Wolborska model indicates the kinetic coefficient of the mass transfer. Then, it shows a concordance with the Clark parameter, in the increasing tendency of the pore volumes injected to each packed sand bed. It presents a higher mass transfer between sand and the fines effluent in the third treatment (800 mg·L^−1^ CTAB adsorbed), which offers a higher retention of fines.

### 4.5. Estimation of the Critical Rate for Fines Migration

**Figure 14:**
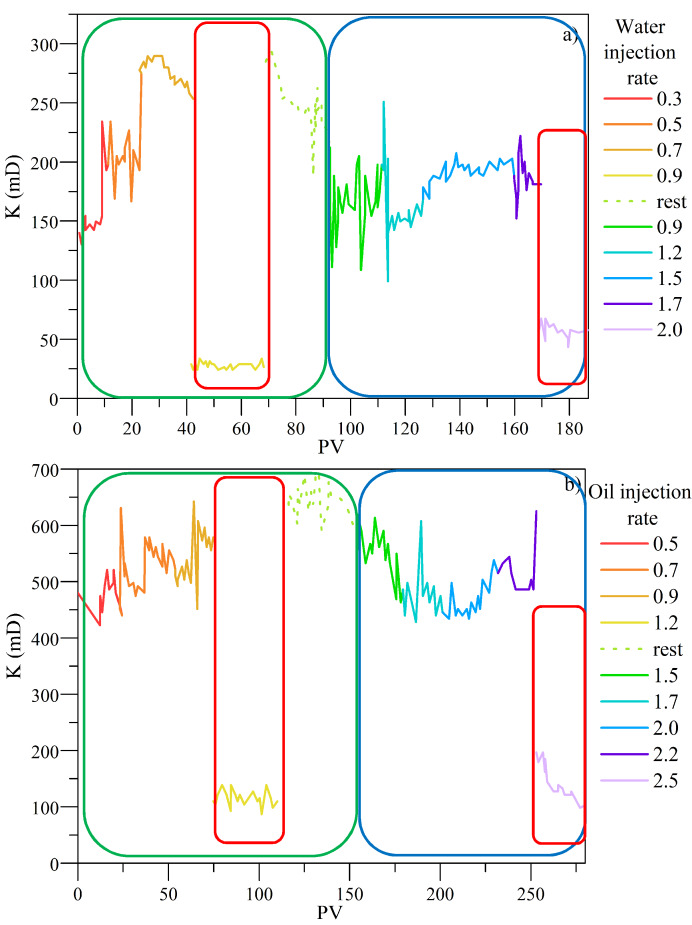
Critical rate to (**a**) water—before (green box) and after treatment (blue box) and (**b**) oil—before (green box) and after treatment (blue box) composed of MgO-based nanofluid with 800 mg·L^−1^ of CTAB adsorbed.

Figure 14 summarizes the permeability for oil and water of the system in the absence and in the presence of the nanofluid containing MgO nanoparticles with 800 mg·L^−1^ CTAB adsorbed. From the results, it was observed a decrease in water and oil permeabilities at 0.7 mL·min^−1^ and 0.9 mL·min^−1^, respectively, in the absence of the MgO-based nanofluid. By contrast, with the treatment injection, critical rate increased in 142.8%, and 144.4% through water and oil flow, respectively, showing a higher improvement in this phenomenon than other materials [23,68]. According to these results, for both environments flow, the treatment stabilized the fines particles, increasing the last rate in which the system did not present a drop in the permeability of the phases. This corroborates under dynamic conditions the successful behavior of the optimized nanocomposite.

## 5. Conclusions

Using a simple one-step method, nanostructured magnesium oxide powders with sizes in the range of 86.2–11.5 nm were obtained. The size of magnesium oxide particles decreased with increasing the concentration of the precursor. For the 2000 mg·L^−1^ of nanoparticles-based dosage treatment, the amount of porous volumes injected increased up to more than two times before reaching saturation, similar to the 2000 mg·L^−1^ treatment of CTAB. A synergistic effect between the MgO and CTAB nanoparticles was visualized in the increase of the medium capacity for inhibiting the fines migration based on the laboratory tests made. The efficiency of this treatment increased up to 464% higher than the medium without a treatment applied.

The statistical analysis with the mixture design experiments shows that the optimal concentration for the treatment was 98.53% deionized water, 0.73% of magnesium oxide nanoparticles, and 0.74% of CTAB. Additionally, a similar affinity to inhibit fines migration with the nanoparticles than the treatment with only CTAB was visualizeed. The adsorption isotherms were developed at three different temperatures of 25, 35, and 45 °C to evaluate the interaction between both compounds, finding the highest capacity to keep the surfactant adsorbed on the nanoparticle surface at the lowest temperature. Additionally, by the desorption tests, the maximum desorption percentage was 25%. The retention tests for the treatments with the CTAB adsorbed MgO nanoparticles (System II) reached a better performance than the treatments with the CTAB suspended in solution (System I). The breakthrough curves show that the efficiency of this treatment increased by 654% higher than the medium without a treatment apply. Regarding the core flooding test, the critical rate increased in 142.8%, and 144.4% for water and oil flow in the presence of the nanofluid under dynamic conditions. It presents MgO nanoparticles functionalized with CTAB as the best alternative for the fines migration problem.

## Figures and Tables

**Table 1:** Estimated mean particle size by DLS (*dp*_50_), estimated surface area (*S*_BET_), point of zero charge (pH_pzc_), and zeta potential at pH = 7 (*ζ*_pH = 7_) of the three synthesized MgO nanoparticles at 25 °C.

Nanoparticle	Concentration Ratio[MgNO_3_·6H_2_O]/[NaOH]	*dp*_50_ (nm)	*S*_BET_ (m^2^·g^−1^)	pH_pzc_	*ζ*_pH = 7_ (mV)
M11	1.2	11.4	147.1	11.7	15.4
M42	0.8	42.8	39.2	11.4	11.2
M86	0.4	86.2	19.4	11.0	11.3

**Table 2:** Calculated parameters of the SLE model for adsorption isotherms of CTAB onto MgO nanoparticles at a fixed concentration of nanoparticles (1200 mg·L^−1^) at 25, 35, and 45 °C.

*T* (°C)	*H* (mg∙g^−1^)	*K* (g∙g^-1^) × 10^−2^	*N*_m_ (g∙g^−1^)	% *RMSE*	*R* ^2^
25	0.91	100.01	14.01	2.51	0.98
35	1.09	100.23	13.14	2.46	0.99
45	1.29	100.26	12.74	2.89	0.99

**Table 3:** Response parameters of Clark [48] and Wolborska [49] models for treatments evaluated varying the concentration of MgO nanoparticles-M11, CTAB, and deionized water.

Evaluated Treatment	Wolborska Model		Clark Model	
	*β* (min^−1^)	% *RSM*	*N*_0_ (mg·L^−1^)	% *RSM*
Blank	0.422	9.4	0.02	9.9
1000 mg·L^−1^ M11	0.611	9.4	0.17	8.8
2000 mg·L^−1^ M11	0.610	9.8	0.19	9.9
1000 mg·L^−1^ CTAB	0.602	7.7	0.16	9.4
2000 mg·L^−1^ CTAB	0.628	8.3	0.20	6.7
660 mg·L^−1^ M11 /CTAB	0.631	9.5	0.39	9.9
1000 mg·L^−1^ M11/CTAB	0.639	7.9	0.26	8.0

**Table 4:** Calculated parameters of the special cubic model using the Clark model for the mixtures of MgO nanoparticles and CTAB evaluated in system I.

*β*_1_	*β*_2_	*β*_3_	*β*_12_	*β*_13_	*β*_23_	*β*_123_	*R* ^2^
0.018258	0.18986	0.15398	0.097124	0.27718	0.36440	5.14033	1

**Table 5:** Answer parameters of Clark [48] and Wolborska [49] models for treatments evaluated varying the concentration of adsorbed CTAB.

Evaluated Treatment	Wolborska Model		Clark Model	
	*β* (min^−1^)	% *RSM*	*N*_0_ (mg·L^−1^)	% *RSM*
247 mg·L^−1^ CTAB	0.83	6.9	0.22	7.3
515 mg·L^−1^ CTAB	1.40	7.8	0.35	2.1
800 mg·L^−1^ CTAB	1.60	2.8	0.48	8.8

## Supplementary Materials

The following are available online at https://www.mdpi.com/2079-4991/10/5/928/s1. Figure S1. TEM analysis for the MgO nanoparticles samples (a) M11, (b) M42, and (c) M86, where the number of the nomenclature refers to the hydrodynamic diameter (nm) found for each particle by DLS technique at 25 °C. Figure S2. Zeta Potential of the three synthesized MgO nanoparticles at 25 °C. Table S1. Properties of porous media employed for the oil recovery experiments.
