# Supplementary material for: Development of Nanofluids for the Inhibition of Formation Damage Caused by Fines Migration: Effect of the Interaction of Quaternary Amine (CTAB) and MgO Nanoparticles
Source: Nanomaterials (Basel). 2020 May 11;10(5):928. doi: 10.3390/nano10050928 (PMC7279548; doi:10.3390/nano10050928)
Supplement: Supplementary file 1 [file nanomaterials-10-00928-s001.pdf]

Supplementary Material

Development of Nanofluids for the Inhibition of Formation Damage Caused by Fines Migration: Effect of the interaction of Quaternary Amine (CTAB) and MgO Nanoparticles

Rebeka Díez †, Oscar E. Medina †, Lady J. Giraldo, Farid B. Cortés *, and Camilo A. Franco *

Research Group on Surface Phenomena - Michael Polanyi, Faculty of Mines, National University of Colombia, 050034 Medellín, Colombia; rdiezb@unal.edu.co (R.D.); oemedinae@unal.edu.co (O.E.M.); ljgiraldop@unal.edu.co (L.J.G.)

* Correspondence: fbcortes@unal.edu.co (F.B.C.); caafrancoar@unal.edu.co (C.A.F.); Tel.: +574-4255137 (F.B.C)

† Authors contributed equally to this manuscript

a)

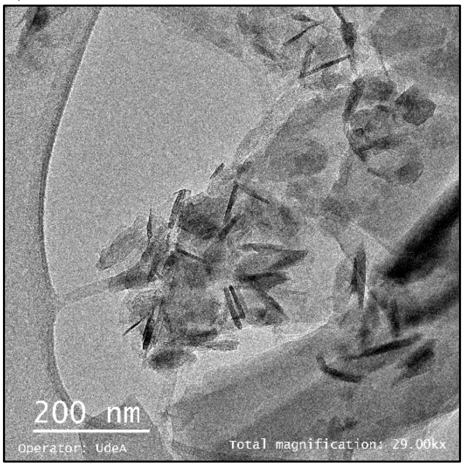

b)

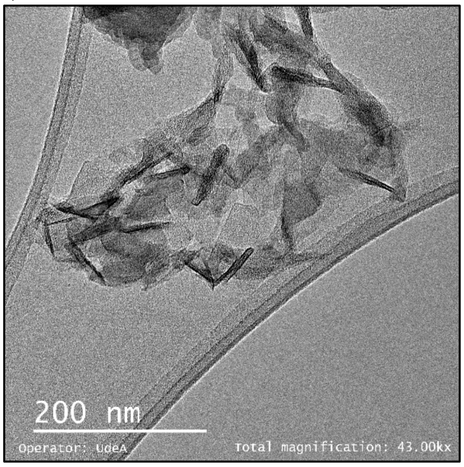

c)

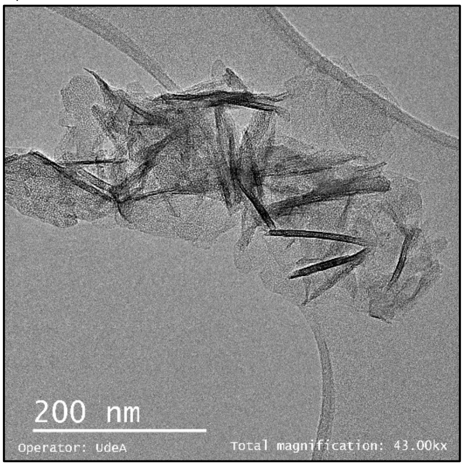

Figure S1. TEM analysis for the MgO nanoparticles samples a). M11 b). M42 and c). M86, where the number of the nomenclature refers to the hydrodynamic diameter (nm) found for each particle by DLS technique at 25°C.

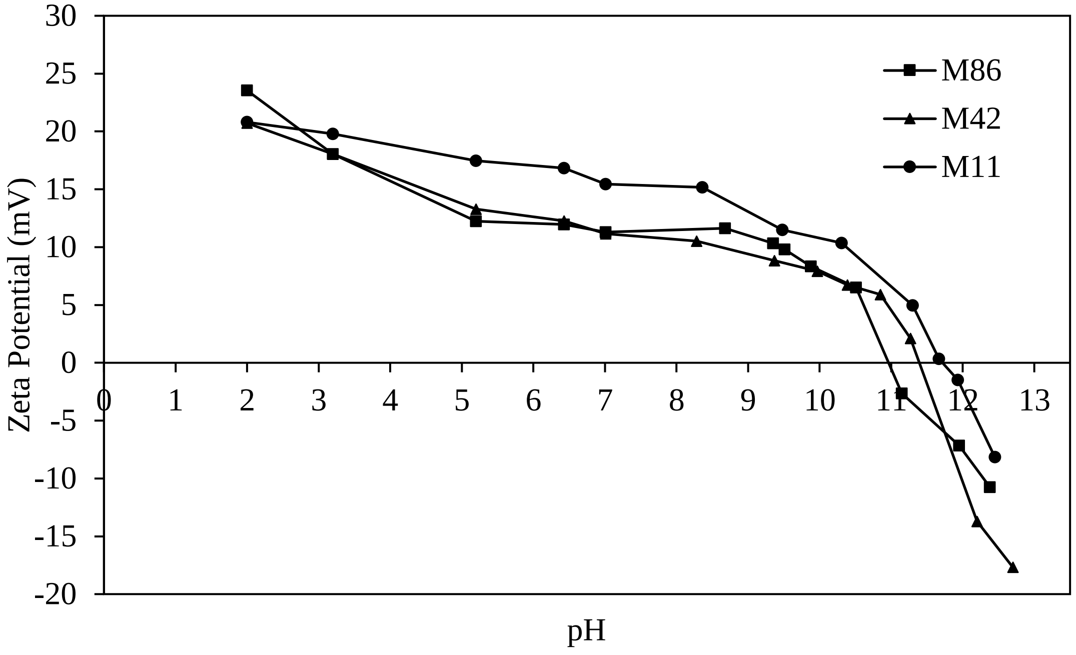

Figure S2. Zeta Potential of the three synthesized MgO nanoparticles at 25ºC.

Table S1. Properties of porous media employed for the oil recovery experiments.

Property	Value
Mineralogy	99% Silica
Length	4.15 cm
Diameter	3.81 cm
Porosity	11.1 %
Porous Volume	5.22 cm^3
Water initial relative permeability	140
Oil initial relative permeability	445
